# “I'm too old for this!”: A prospective, multilevel study of job characteristics, age, and turnover intention

**DOI:** 10.3389/fpsyg.2022.1015313

**Published:** 2022-11-24

**Authors:** Jan Olav Christensen, Stein Knardahl

**Affiliations:** Group of Work Psychology and Physiology, National Institute of Occupational Health, Oslo, Norway

**Keywords:** turnover, job characteristics, psychosocial work environment, aging, job design

## Abstract

**Introduction:**

Deciding to leave a job is often foreshadowed by burgeoning job dissatisfaction, which is in turn often attributed to characteristics of the job and work environment. However, while we know that job characteristics influence job satisfaction, health, and motivation, their associations with turnover intention is less clear. Moreover, despite aging workforces, an understanding of how working conditions influence workers across the lifespan is lacking. Therefore, drawing on job design theories and bridging turnover- and aging research, we studied 15 specific job characteristics to determine whether they predicted turnover intentions, and whether the predictive value was modified by age.

**Methods:**

Data were collected from various public and private enterprises in Norway. Moderated multilevel regressions were conducted cross-sectionally (*N* = 12,485) and prospectively over 2 years (*N* = 5,504).

**Results:**

Most work factors were associated with turnover intention at both the individual and work unit levels. *A social climate of support, trust, and encouragement* was most strongly inversely associated with turnover intentions, while role conflict was most strongly positively associated with turnover intentions. *Organizational climate, leadership styles*, and *job control* were more important with age while *job demands, predictability* and *role stressors* were more important to younger workers. Ten individual level- and four work-unit level factors predicted turnover intentions prospectively, suggesting turnover intentions due to poor working conditions persisted in employees that did not quit.

**Discussion:**

Our results highlight several specific, modifiable job characteristics that are likely to affect turnover intentions, and the impact of certain factors specifically for older workers.

## 1. Introduction

For more than a century, researchers have sought to understand conditions that lead employees to leave organizations (for a comprehensive review, see Hom et al., 2017). The continued interest in this topic reflects a recognition of the organizational cost of turnover, which includes production disruption and loss of key employees as well as organizational memory (Shaw et al., 2005; Hausknecht et al., 2009; Heavey et al., 2013; Park and Shaw, 2013). Hiring and replacement expenses are often higher than the annual salary of the position being filled (Cascio, 2006; Allen et al., 2010). For highly qualified staff with skill sets in high demand, unemployment rates and labor market competition have limited impact on turnover (Trevor, 2001). Moreover, even when actual turnover is low, high rates of turnover intentions may disrupt both organizational functioning and the wellbeing of individuals that want to quit when it is not possible (“trapped stayers,” Hom et al., 2012).

*Work design* has been studied for decades due to it's potential of “enriching” jobs, i.e., altering their characteristics to make them more motivating and enjoyable to the worker and in turn improve performance (Hackman and Oldham, 1980). Work design can be defined as “the content and organization of one's work tasks, activities, relationships, and responsibilities” (Parker, 2014, p. 662), potentially encompassing a broad range of characteristics of both the content and context of work. One of the most influential theories of job characteristics, the “job characteristics model”, states that there are five core dimensions that characterize jobs and determine psychological states that influence motivation, performance, satisfaction, and turnover (Hackman and Oldham, 1976). These core characteristics are skill variety (degree to which the job requires different skills, giving the opportunity to utilize one's skills and talents), task identity (degree to which the job requires finishing a “whole,” identifiable piece of work), task significance (degree to which the task is perceived as important and consequential), autonomy (degree of freedom and independence in planning and executing tasks), and feedback (information about the effectiveness of one's performance). While the job characteristics model has been pivotal, scholars have criticized it for not being comprehensive and have suggested that it is overused by researchers, leading to the negligence of numerous important and more specific characteristics of jobs (Morgeson and Humphrey, 2006).

Work design contributes to a wide range of worker outcomes, such as performance, pain, mental distress, positive affect, retirement decisions, and even mortality (for systematic reviews, see Kraatz et al., 2013; Browne et al., 2019; Taouk et al., 2020; Knight and Parker, 2021). While it may seem intuitively appealing, then, that work characteristics are essential to turnover decisions, this assumption has received relatively little attention. Turnover research has often focused on precursors that are more proximate, i.e., temporally closer to turnover decisions, most commonly job satisfaction and organizational commitment (Hom et al., 2017), both of which may be seen as consequences of job characteristics. There are examples of studies that have explored the relationship of working conditions with job dissatisfaction and subsequent quitting (see e.g., Böckerman and Ilmakunnas, 2009 for a study utilizing registers in Finland to study actual turnover). Nevertheless, a 2022 comprehensive, systematic review revealed that only 13% of studies of turnover and turnover intention have examined antecedents referring to “aspects of the job,” a category that incudes job characteristics but also factors such as pay and job security (Bolt et al., 2022). Moreover, the studies that have been conducted have generally been limited by modest sample sizes, single-level cross-sectional designs, restrictions to specific occupations, and a majority are geographically restricted to North America (Kim and Kao, 2014; Bolt et al., 2022).

As pointed out by Rubenstein et al. (2018), studies concerning “aspects of the job” suggest interesting avenues for future studies, as they imply that “managers can make active efforts to reduce an individual's turnover likelihood rather than assuming such decisions are made purely on the basis of general dissatisfaction or dispositional factors” (p. 15). That is, job characteristics are amenable to planned change and therefore studies of such factors provide directly applicable knowledge. Examining a comprehensive range of specific work factors should aid decision-makers and practitioners in prioritizing factors for interventions (e.g., job enrichment efforts) and policy formation. Previous work design studies have typically included fewer factors, often measured as relatively broad dimensions, to test predictions based on established theories at a relatively high level of abstraction (e.g., “job demands” or “resources”). While this is important for theory development, the value for designing interventions in practice should be enhanced by knowledge of specific work factors. The present study sought to address this, and also concurs with recent calls for more exploratory approaches in organizational psychology (Spector and Pindek, 2016; Spector, 2017). Specifically, Spector and coworkers have cautioned against an overreliance in organizational sciences on hypothetico-deductive approaches, where testing hypotheses derived from large scale theories is seen as necessary and inductive exploration is deemed inappropriate and somehow inferior. According to Spector (2017), this may prevent new discovery, and has even “created unintended challenges to research integrity of confirmation bias, p- hacking, HARKing, and the chrysalis effect” (p. 1). With regard to the associations between work characteristics and turnover intentions, the current study adopts elements of an exploratory approach by testing a broad range of specific work factors, each of which are theoretically justified and described below, but not subsumed under a general theoretical framework.

In view of the aforementioned limitations of previous research, the first central aim of the present study is to extend the knowledge base by examining both cross-sectional and prospective, multilevel associations of a comprehensive selection of job characteristics with turnover and turnover intentions. One of the original motivations for the job characteristics model was to address “the paucity of conceptual tools that are directly useful in guiding the implementation and evaluation of work redesign projects” (Hackman and Oldham, 1976, p. 251). Importantly, the included work factors are relatively specific in order to be useful to practitioners.

Norway, like the other Nordic countries, is characterized by generally good working conditions and a generous welfare system. However, an aging population challenges the sustainability of this system. In order to meet this challenge, and also because work can be intrinsically meaningful and salutogenic under the right conditions, it is necessary to gain further knowledge about factors that promote the desire in workers to remain in their current job. More generally, the challenge of keeping all members of an increasingly age-diverse workforce engaged and productive has stimulated scholars to recommend more *age-differentiated* human resource management (Li et al., 2011; Thielgen et al., 2015; Cox et al., 2019). Although job enrichment may be beneficial to most workers, lifespan aging theories (Baltes and Baltes, 1990; Carstensen et al., 1999) suggest that workers of different ages may require different modifications of work content and -environment for it to actually be enriching. In short, lifespan theories suggest that systematic changes across the life-span can determine several adaptive processes in workers, which have a bearing on priorities, choices, behavior, and responses to the social environment, including the that of work. As workers spend a significant proportion of their lives working, and it is an important part of life for most, the adaptive processes that are associated with age and aging may be of significance to explain how working conditions affect workers (Fazi et al., 2019). Thus, the second central aim for the current study was to elucidate whether the impact of work characteristics on the intention to quit varies with employee age.

### 1.1. The study of turnover intentions: Why and when employees decide to quit

Most early turnover studies were concerned with attitudes, like dissatisfaction with the job, as antecedents of leaving a job. The influential theory of voluntary turnover by March and Simon (1958) posited that the choice to leave an organization is an interaction between the individual's perception of the desirability of leaving and the perceived ease of movement. Later, Porter et al. (1974) showed that organizational commitment, i.e., the extent to which the individual identifies with the organization, was as important as satisfaction with the job itself. Further elucidating the complexities of the turnover process, Mobley (1977) published a model that described seven sequential stages from initial dissatisfaction to actual turnover.

Shifting from the exclusive focus on proximal precursors, Lee and Mitchell (1994) put forward the *unfolding model of turnover* proposing a variety of distal and proximal disruptive events, such as job offers from other employers, events that violate the employee's ethics or goals, or personal events, playing a major role in turnover. These events were denoted “shocks” and decision “paths” were specified to describe the process from such “shocks” to a final quitting decision. The model took into account factors that strengthen the motivation to stay in the job, which may prevent turnover even after “shocks” occur, labeled *job embeddedness* (Lee et al., 2004, 2017). Such factors may for instance pertain to valued aspects of job content or -environment that diminish the likelihood of employees leaving in spite of attractive alternatives.

Specifically focusing on exogenous factors in the work situation, Mobley et al. (1979) reviewed factors that may explain the *initiation* of the turnover process, including a variety of “organizational and work environment factors” (e.g., role pressures and social climate) and “job content factors” (e.g., workload and autonomy). These factors can be seen as more contextual or distal to the actual turnover behavior or -attitude. During more recent years, some studies have demonstrated effects of task- and job characteristics, working conditions, social interaction and “quality of work” on turnover and turnover intentions, (e.g., Houkes et al., 2001; Huang et al., 2007; Podsakoff et al., 2007; Arnoux-Nicolas et al., 2016; Salin and Notelaers, 2017). Nevertheless, turnover research as a whole has continued to primarily elucidate individual predictors (e.g., attitudes, age, personality, motives) and exchange aspects of the job (e.g., workload and salary), while work content and contextual predictors (e.g., organizational climate and -support) remain under-explored (Holtom et al., 2008; Hom et al., 2017; Rubenstein et al., 2018).

Allen et al. (2010) discussed five common misconceptions about employee turnover, which illustrate a tendency to still overlook the influence of working conditions. One belief is that people quit because of pay, when in fact pay level and pay satisfaction are weak predictors of decisions to quit (Allen et al., 2010). Moreover, while much research has examined job satisfaction and a common assumption still is that “people quit because they are dissatisfied with their jobs,” it seems that job dissatisfaction is the main factor in less than half of turnover decisions (Lee et al., 1999).

Markey et al. (2012) studied the “quality of the work environment” (QWE), incorporating aspects of the psychosocial work environment, and found that employees were less likely to intend to quit when appraising the QWE positively. Hence, they concluded that the QWE should be targeted in policy formation in order to influence quitting intentions. Kim and Kao (2014) conducted a meta-analysis of cross-sectional studies investigating correlates of turnover intention among U.S. child welfare workers. They concluded that factors of the work environment, such as support from supervisors or co-workers, role conflict, role ambiguity, inclusion, fairness perceptions, organizational climate, autonomy, and job demands, were associated with turnover intention (Kim and Kao, 2014). However, this and most previous turnover research was cross-sectional and conducted with small, homogenous samples (Hayes et al., 2006). Thus, results may be influenced by selection biases and reverse causality due to cognitive biases from employees minimizing cognitive dissonance and adjusting their judgment of work characteristics to align with and support their decision to quit.

### 1.2. Why the work experience and its effect on turnover decisions may change with age

Recent years have seen an increased interest in investigating age differences in the impact of job characteristics (Fazi et al., 2019). In particular, two *life-span development approaches* have been prominent in this endeavor, namely *Selection, Optimization, and Compensation* (SOC) theory (Baltes and Baltes, 1990) and *Socio-Emotional Selectivity* (SES) theory (Carstensen et al., 1999). Both theories describe behavioral and motivational adaptations that individuals tend to make in response to the process of aging, which entails both losses that must be coped with and gains that can be utilized to promote wellbeing.

SOC theory describes how workers aim to reduce losses and maximize gains by *selecting* suitable goals, *optimizing* them, and *compensating* for age-related losses (Baltes and Baltes, 1990). Such strategies are used throughout life to adapt to and cope with environmental demands in a way that is believed to be best at one's current age. Selection and prioritization of specific goals is necessary so more attention can be devoted to goals that are attainable and meaningful, rather than dividing limited resources across all possible goals. Optimization enables individuals to focus efforts and resources on selected goals in order to be likely to achieve them. Finally, compensation involves strategies that offset age-related declines or losses to maintain a certain level of performance. SOC theory also suggests that aging entails a shift from *promotion* (i.e., gaining competencies, growth, and development) to *prevention* (i.e., avoiding losses while maintaining resources and security; Ebner et al., 2006). Empirical evidence has suggested that SOC behaviors can foster adaptive processes that slow down age-related declines of work ability (Weigl et al., 2013), job satisfaction (Schmitt et al., 2012), wellbeing (Wiese et al., 2002), and job performance (Bajor and Baltes, 2003).

SES theory is centered on the selection of goals, the main proposition being that an individual's *perception of time* influences social goal pursuits (Carstensen et al., 1999). The notion of a *future time perspective* and how it changes over time is essential to this notion. Younger individuals perceive time as more open-ended, implying that knowledge-related goals (i.e., efforts to increase knowledge and resources) are prioritized. As the individual ages, emotion-related goals are increasingly prioritized, since time is perceived as more limited and the perspective is oriented toward present experience. A central implication of this may be that with age, workers increasingly focus on social and emotion-related goals, implying that higher value is placed on positive work experiences rather than enduring less positive experiences for the sake of future gains. SES has been used to explain various goals and motives for workplace social interactions across the life span (Kooij et al., 2011).

Another lifespan theory, pertaining specifically to the concept of control, is the *life-span theory of control* (Heckhausen and Schulz, 1995; Heckhausen et al., 2010). According to this theory the use of *primary* vs. *secondary* control strategies increases with age. Primary control strategies seek to alter the external world in order to resolve a challenge (problem-focused coping; Lazarus and Folkman, 1984), whereas secondary control strategies involve adaptation by altering oneself. This suggests that empowerment and job control would be particularly valuable with age, as they give opportunities to alter work processes and -conditions. Consistent with this notion, but more generally, Yaldiz et al. (2018) argued that beneficial job aspects should be most beneficial to older workers.

Drawing on the two main life-span development approaches (i.e., SOC and SES), Truxillo et al. (2012) formulated hypotheses about how effects of job design depend on age. A number of specific propositions were developed suggesting that (1) job characteristics that match the value systems specific to an age group would be more important to that group and a greater effect would be expected than for other age groups, and (2) that characteristics of the job that align with SOC strategies would be more important to older workers (e.g., Bal et al., 2013). Some studies have tested propositions derived from Truxillo et al. (2012). Zaniboni et al. (2013) studied age-differential effects of task- and skill variety on burnout and turnover, finding that older workers were less likely to report turnover intentions when experiencing greater skill variety. Moreover, Zaniboni et al. (2016) found that job autonomy had a greater effect on job satisfaction and mental health for older workers.

Although lifespan theories render age-differential effects of job- and work environment characteristics plausible, specific predictions seem less straightforward to derive. Illustrative of this, a meta-analysis by Ng and Feldman (2015) tested two competing hypotheses regarding the effect of autonomy for older workers and found that moderation effects of age depended on the outcome studied. That is, the effects of autonomy were stronger for older workers for job self-efficacy and job performance, but weaker for job satisfaction and affective commitment (Ng and Feldman, 2015).

Quitting one's current job is one possible response to adverse job characteristics and low job satisfaction. Aging workers may consider this option an opportunity to select an alternative job that optimizes their skills and experience and compensates for sub-optimal skills. Furthermore, as the use of primary control strategies increases with age, they may be less likely to cope by changing themselves and more likely to change jobs to improve person-job fit. Moreover, SES theory suggests that employees will value emotional-regulation goals and positive work experiences more with age, and knowledge acquisition and skill-building less (Carstensen et al., 1999). Thus, when the work environment is positive and satisfactory, older workers may emphasize this more than younger workers. They may value social relations, organizational support, a positive perceived context, and the opportunity to draw on existing skills over further skill development and career progress.

### 1.3. Job- and work environment characteristics analyzed in the present study

Mounting evidence suggests work can influence attitudes and wellbeing differently with age. However, which specific work factors may be more important to older workers remains largely unknown. Hence, the present research studied a comprehensive range of specific work factors. This choice also aligned with the aim to enhance the relevance to practical work environment improvement, where targeting multiple specific factors is usually necessary. The range of factors included task level factors, leadership styles and social and relational factors, and pertained to the level of the individual, the work group, and the organization.

Grounded in the job design theories each of the included factors originated from, we expected an impact of these specific work factors on employee turnover and turnover intentions. Furthermore, grounded in the SOC and SES lifespan theories of aging, and the life-span theory of control, we expected stronger associations with age. All hypotheses pertain to cross-sectional effects as well as prospective effects over a period of 2 years.

In the following, a brief rationale is presented for each of the work characteristics.

#### 1.3.1. Job demands

In his seminal 1,979 article Robert Karasek defined job demands as “stress sources (stressors), such as work load demands, present in the work environment” (Karasek, 1979, p. 287). Others have denoted demands as “all those occurrences, circumstances, and conditions in the workplace that put pressure on the individual” (Dallner, 1997). Hence, job demands is an unspecific higher-order dimension, where some aspects can be obviously external to the individual (e.g., a number of units to be manufactured, a specific deadline) while others are inherently subjective (e.g., task difficulty). However, demands are typically measured by instruments referring to quantifiable aspects such as time pressure and amount of work (Karasek et al., 1998). The present study included such *quantitative* demands as well as *decisional* demands, a *qualitative* demand referring to attentional requirements and complex decision making demands (Lindström et al., 1997).

SOC theory suggests older workers select and optimize tasks they already master, and compensate for decreased fluid abilities (i.e., solving novel reasoning problems) with superior crystallized abilities (i.e., relating previously learned concepts to each other). Hence, they may prefer not to take on new tasks or a higher workload, but to focus on tasks they already master, which showcase their accumulated experience and skills. Furthermore, SES theory would suggest that younger workers have more to gain by accepting a high workload, allowing them to build and accumulate skills and competence. Bouville et al. (2018) found that although job demands did not exhibit a main effect, high job demands were related to sickness absence for older workers.

Hypothesis 1a: Job demands (quantitative demands and decision demands) are associated with turnover intention
Hypothesis 1b: The association of job demands with turnover intention increases with age

#### 1.3.2. Role expectations

Job demands are attached to *roles* designating criteria of desirable behaviors. The breakdown of communication of role expectations may have adverse consequences. The current study elucidated effects of *role ambiguity, role conflict* (Kahn et al., 1964; Beehr et al., 1976), and *work-life conflict*. Ambiguity refers to a situation where roles lack clear definition, where employees are unsure of what is expected and what the job content is. Role conflict is the result of two or more expectations being incompatible, either within one job role, between multiple roles one person possesses (e.g., at work and in private life), or between work and personal values and norms (Rizzo et al., 1970). Since role conflict is considered a job demand in the frequently employed Job Content Questionnaire (JCQ; Karasek et al., 1998), it is included (albeit implicitly) in much research connecting psychological work characteristics with health, motivation, and wellbeing.

Cross-sectional studies have found role conflict and -ambiguity to be associated with turnover intentions (Kim and Kao, 2014). A recent meta-analysis reported that role expectations were associated with affective organizational commitment, but not continuance commitment, implying that role conflict and -ambiguity may affect the desire to stay even if the need to stay persists (Morrissette and Kisamore, 2020).

Older workers may be less accepting of conditions entailing ambiguous and conflicting expectations that call for high levels of fluid abilities and accommodation of existing skill sets. Moreover, the life-span theory of control may suggest older workers are less likely and willing to change themselves in order to adapt to such a working situation, and may rather consider changing jobs to improve the situation. This notion is common to all the factors investigated herein.

Hypothesis 2a: Role conflict, role ambiguity, and work-life conflict are associated with turnover intentions
Hypothesis 2b: The associations of role conflict, role ambiguity, and work-life conflict with turnover intention increase with age

#### 1.3.3. Job control

*Job control* is also a dimension that encompasses several factors. Important aspects are the opportunities to influence planning and decision-making relevant to one's job tasks (Dallner et al., 2000). “Job autonomy” was defined by Hackman and Oldham (1980, p. 79) as “the degree to which the job provides substantial freedom, independence, and discretion to the individual in scheduling the work and in determining the procedures to be used in carrying it out.” The Job strain model operationally defines job control by two separate factors—skill discretion and decision authority (Karasek, 1979; Karasek et al., 1998). Skill discretion refers to the opportunity to utilize one's skills and abilities, while decision authority pertains to freedom to make decisions, to influence and to regulate aspects of one's work such as pacing, breaks, and working hours.

Truxillo et al. (2012) suggested that job autonomy may be especially beneficial for older workers as it allows them to craft the job to optimize with regard to their abilities and compensate for the lack of certain abilities. More freedom in the job may be more useful to older employees as they can adapt the job to fit existing skills and competence, whereas for younger employees it may be more important to have clear directives and direction for the future.

Hypothesis 3a: Job control (control over decisions and work pacing) is inversely associated with turnover intention
Hypothesis 3b: The inverse association of job control with turnover intention increases with age

#### 1.3.4. Positive challenges

*Positive challenges* refers to whether the worker perceives existing skills and knowledge as useful, and whether work is meaningful and challenging in a positive way. Positive challenges at work have been found to predict low mental distress and high positive affect (Finne et al., 2016), and to correlate with job involvement and -satisfaction (Dallner et al., 2000).

Positive challenges should be particularly important to older workers, which may focus on the present and select situations that produce positive emotions while avoiding tasks that merely accumulate experience and skills.

Hypothesis 4a: Positive challenges are inversely associated with turnover intention
Hypothesis 4b: The inverse association of positive challenges with turnover intention increases with age

#### 1.3.5. Predictability

Predictability in life is important, noted perhaps most famously by Maslow (1943), referring to the need for safety. As the rate of change in work life seems to accelerate, maintaining a reasonable level of stability and predictability may seem challenging to many. For the current study both short term (1 month) and long term (2 years) predictability were assessed. Short-term predictability regards the opportunity to generate realistic expectations about work events, i.e., what tasks, coworkers, and superiors to expect, while long-term predictability refers to expectations of one's employability in the future (Dallner et al., 2000).

Uncertainty may result in hyper-vigilance and sustained psychological and physiological arousal, which influences wellbeing. However, in spite of the ubiquity of rapid technological and societal advances, occupational health- and organizational psychology research seems to have largely neglected predictability of job tasks (Dallner et al., 2000). Job insecurity, which is one aspect of long-term predictability, is more extensively studied (see e.g., Sverke et al., 2002).

SOC theory implies that older workers might be more appreciative of predictability and stability, which provides the opportunity to execute and optimize already honed skills rather than acquiring new ones to adapt to new situations. Moreover, the increased use of primary control strategies imply that a work situation that demands ongoing adaptation and self-adjustment will be less attractive with age.

Hypothesis 5a: Predictability (short term and long term) is inversely associated with turnover intention
Hypothesis 5b: The inverse association of predictability with turnover intention increases with age

#### 1.3.6. Organizational climate

Organizational climate refers to employees' perceptions of trust, support, innovation, recognition, and fairness in the organization (Taylor and Bowers, 1972; Moran and Volkwein, 1992; Lindström et al., 1997). In the present study two aspects were considered; *social climate* and *human resource primacy*.

Social climate entails the degree of support from within the work unit. Much previous research has focused on social support defined by supportive behaviors or availability of advice, assistance, feedback, and empathy (House et al., 1982; Thoits, 2011), especially in connection with the Job strain model, which posits the moderative effect of support on the effect of strain (Johnson and Hall, 1988).

Human resource primacy, on the other hand, refers to “the extent to which the climate, as reflected in the organization's practices, is one which asserts that people are among the organization's most important assets” (Michaelsen, 1973, p. 18). Hence, human resource primacy highlights the role of top management and the degree to which the prioritization of employee health and well-being is institutionalized and known to the employees.

Again, SES theory posits that older individuals are more oriented toward the present than the future, prioritizing emotional regulation and positive experiences. Organizational climate and -support may be of particular importance with age as one may attribute more value to working for an organization with a supportive climate that commits to fair treatment, employee appreciation and wellbeing.

Hypothesis 6a: Social climate and human resource primacy are inversely associated with turnover intentions
Hypothesis 6b: The inverse associations of social climate and human resource primacy with turnover intentions increase with age

#### 1.3.7. Leadership styles

Leadership is closely tied to many other work factors, as leaders and managers by definition have the power to influence ways of working. For instance, leaders may differ in the extent to which they organize production processes to facilitate employee autonomy. *Empowering leadership* refers to the degree to which employees are encouraged by superiors to take part in important decisions, to express opinions, and to develop skills (as opposed to being delegated specific tasks) (Dallner et al., 2000). Empowering leadership may provide opportunities to satisfy the need for autonomy, which is considered a basic psychological need (Ryan and Deci, 2017). However, effects can also be mediated by the practical adjustments that autonomy and self-directness allow, which older employees may be more likely to utilize since they have accumulated more experience and know-how.

As mentioned above, employee perceptions of fairness and support are important aspects of the organizational climate. *Superior support* and *fair leadership* refer to ways in which leaders contribute to this. Support refers to the instrumental and emotional support the closest supervisor provides, while fair leadership pertains to the degree to which the leader treats employees fairly and equally and distributes tasks fairly (Dallner et al., 2000). Both aspects of leader behavior have been linked to lower levels of mental distress and higher positive affect (Finne et al., 2016). Again, prioritization of emotional regulation and positive work experiences combined with the use of primary control strategies would suggest that, overall, quitting is an option that is more salient to older employees if and when the leader is not empowering, supportive and fair.

Hypothesis 7a: Empowering-, supportive-, and fair leadership styles are inversely associated with turnover intentions
Hypothesis 7b: The inverse associations of empowering-, supportive-, and fair leadership styles with turnover intentions increase with age

## 2. Methods and materials

### 2.1. Design and procedure

The study was prospective with two waves. Average follow-up period was 24 months (17–36 months). Previous meta-analyses have suggested a 2–3 years time-lag to be appropriate for detecting occupational stressor-strain associations (Ford et al., 2014).

The study was based on an ongoing project that includes work environment surveys in Norway. Data collection has been ongoing since 2004. The aim of the project is to comprehensively study various characteristics of working conditions and their relationship with health, wellbeing, work ability, sick leave, and attitudes toward work.

Companies participated after contacting the researchers in response to information disseminated by the project web page, or requesting work environment surveys in general. All currently working employees of each company were invited to participate.

Private and public enterprizes participated, representing a variety of sectors and types of companies, such as municipalities, health care, finance, insurance, education, and non-profit. All employees received a letter with information about the survey, informed consent, confidential treatment of responses, ethical considerations, and a personalized code for login to a web questionnaire.

### 2.2. Study population

Two samples were defined for the analyses, a *cross-sectional* sample targeting all employees in companies participating at least once, and a *prospective* sample comprising employees from companies that participated at least twice. The analyses were multilevel, based on work unit exposure levels, i.e., mean levels of work factors within work units. To enhance the reliability, responses were only included from work units where *at least five employees* completed the survey. Group level characteristics are more reliably estimated with more observations within each group, and small group sizes in multilevel models can cause considerable bias (Schunck, 2016).

At baseline (T1) 12,470 (56.5% of eligible) employees returned information for *cross-sectional* analyses. Furthermore, 5,493 (45.2% of eligible) returned information at both time points. Missing data was 1.3% both cross-sectionally and prospectively and considered negligible.

Sample characteristics are given in Table 1. Of the cross-sectional sample, 21% reported some degree of thinking about quitting, while 21.7% reported some degree of actually intending to quit. These numbers were lower for the prospective sample (16.7 and 17.4%, respectively).

**Table 1:** Baseline characteristics of the cross-sectional and prospective study samples (*N* = 12,470 and *N* = 5,493, respectively).

	**Cross-sectional sample**		**Prospective sample**	
	***N*** **(%)**	**Mean (SD)**	***N*** **(%)**	**Mean (SD)**
Age		43.7 (10.7)		44.7 (10.0)
Female	7,091 (56.8)		2,936 (53.3)	
Skill level				
< 10 years	132 (1.1)		44 (0.8)	
10–12 years	4,356 (35.0)		1,979 (36.0)	
13–15 years	3,152 (25.3)		1,345 (24.5)	
>15 years	3,656 (29.3)		1,553 (28.3)	
Managers and unspecified	1,174 (9.4)		572 (10.4)	
Turnover intention		2.17 (1.23)		2.02 (1.16)

### 2.3. Independent variables—Work characteristics

Psychosocial work characteristics were measured with The General Nordic Questionnaire for Psychological and Social Factors at Work (*QPS*_*Nordic*_; Dallner et al., 2000). These factors pertain to tasks, individual work attitudes, and group- and organizational level constructs and originate from theories and models of organizational behavior, work motivation, job satisfaction, job stress, wellbeing, and work-related health, such as Organizational Role Theory (Kahn et al., 1964), the Job Characteristics Model (Hackman and Oldham, 1975), and the Job Strain model (Karasek, 1979). The *QPS*_*Nordic*_ has displayed good psychometric properties in several studies (Dallner et al., 2000; Wännström et al., 2009).

Fifteen scales were studied, as described in the introduction and in Table 2, where cronbach's alpha for all scales are given. Example items for the 15 scales are: Quantitative demands: “Do you have too much to do?,” decision demands: “does your work require complex decisions?,” positive challenges at work: “is your work challenging in a positive way?,” decision control: “can you influence decisions that are important for your work?,” control of work pacing: “can you decide yourself when you are going to take a break?,” role ambiguity: “do you know exactly what is expected of you at work?,” role conflict: “do you receive incompatible requests from two or more people?,” empowering leadership: “does your immediate superior encourage you to participate in important decisions?,” fair leadership: “does your immediate superior treat the workers fairly and equally?”, support from superior: “if needed, can you get support and help with your work from your immediate superior?,” human resource primacy: “to what extent is the management of your organization interested in the health and wellbeing of the personnel?,” social climate: “what is the climate like in your work unit? Encouraging and supportive,” predictability 1 month: “do you know in advance what kind of tasks to expect a month from now?,” predictability 2 years: “do you know what has to be learned and which new skills have to be acquired in order for you to maintain a job that you consider attractive in 2 years?,” work-life conflict: “do the demands of your work interfere with your home and family life?.”

**Table 2:** Means, standard deviations, intraclass correlations, cronbach's α, and pairwise pearson's correlations among study variables within work groups (below the diagonal) and between work groups (above the diagonal) in the cross-sectional sample at baseline.

	**Mean**	**SD**	**ICC1**	**ICC2**	**α**	**Age**	**>50**	**TI**	**QD**	**DD**	**PC**	**DC**	**CWP**	**RA**	**RC**	**EL**	**FL**	**SS**	**HRP**	**SC**	**P1M**	**P2Y**	**WLC**
Age	43.7	10.7	−	−	−	−	0.88^**^	−0.27^**^	0.11^**^	0.01	0.30^**^	0.22^**^	0.21^**^	−0.01	−0.18^**^	0.01	−0.05	−0.08	0.09	−0.01	0.33^**^	−0.04	−0.01
>50	29.2%	−	−	−	−	0.77^**^	−	−0.26^**^	0.11^**^	0.01	0.30^**^	0.17^**^	0.14^**^	−0.03	−0.20^**^	0.00	−0.04	−0.06	0.09	0.00	0.30^**^	0.01	−0.02
TI	2.17	1.23	0.08	0.60	*r* = 0.70	−0.18^**^	−0.18^**^	−	0.24^**^	0.01	−0.40^**^	−0.07	0.20^**^	0.46^**^	0.36^**^	−0.25^**^	−0.37^**^	−0.36^**^	−0.39^**^	−0.42^**^	−0.23^**^	−0.07	0.23^**^
QD	2.93	0.76	0.17	0.78	0.75	−0.03^**^	−0.05^**^	0.14^**^	−	0.33^**^	0.24^**^	0.20	0.26^**^	0.28^**^	0.07	0.13^**^	−0.08	−0.09	0.09	−0.0	0.08	0.12^**^	0.57^**^
DD	3.49	0.71	0.11	0.66	0.63	0.00	−0.02	0.03^**^	0.44^**^	−	0.37^**^	−0.22^**^	−0.30^**^	−0.15^**^	0.26^**^	−0.01	−0.09	−0.10^**^	−0.10^**^	−0.05	−0.11^**^	0.14^**^	0.32^**^
PC	3.98	0.75	0.14	0.72	0.77	0.10^**^	0.07^**^	−0.32^**^	0.13^**^	0.34^**^	−	0.32^**^	0.03	−0.18^**^	−0.30^**^	0.38^**^	0.27^**^	0.27^**^	0.37^**^	0.45^**^	0.32^**^	0.37^**^	0.21^**^
DC	2.97	0.78	0.19	0.79	0.73	0.06^**^	0.03^**^	−0.16^**^	0.00	0.06^**^	0.33^**^	−	0.74^**^	0.29^**^	−0.33^**^	0.49^**^	0.24^**^	0.30^**^	0.48^**^	0.31^**^	0.38^**^	0.27^**^	0.17^**^
CWP	3.22	1.08	0.45	0.93	0.82	0.08^**^	0.04^**^	−0.09^**^	−0.09^**^	−0.11^**^	0.15^**^	0.52^**^	−	0.45^**^	−0.29^**^	0.29^**^	0.07	0.06	0.27^**^	0.11^**^	0.25^**^	0.11^**^	0.17^**^
RA	1.80	0.76	0.15	0.74	0.82	−0.12^**^	−0.10^**^	0.27^**^	0.08^**^	−0.07^**^	−0.28^**^	−0.13^**^	−0.03^**^	−	0.32^**^	−0.10^**^	−0.32^**^	−0.35^**^	−0.23^**^	−0.30^**^	−0.19^**^	−0.04	0.34^**^
RC	2.57	0.80	0.11	0.68	0.71	−0.10^**^	−0.10^**^	0.30^**^	0.35^**^	0.25^**^	−0.13^**^	−0.13^**^	−0.16^**^	0.30^**^	−	−0.38^**^	−0.43^**^	−0.43^**^	−0.56^**^	−0.52^**^	−0.44^**^	−0.14^**^	0.15^**^
EL	3.13	1.02	0.11	0.68	0.87	−0.04^**^	−0.05^**^	−0.26^**^	0.03^**^	0.11^**^	0.33^**^	0.36^**^	0.19^**^	−0.26^**^	−0.20^**^	−	0.68^**^	0.78^**^	0.71^**^	0.58^**^	0.26^**^	0.37^**^	0.05
FL	3.94	0.86	0.10	0.65	0.81	0.00	0.00	−0.31^**^	−0.16^**^	−0.05^**^	0.23^**^	0.23^**^	0.15^**^	−0.32^**^	−0.37^**^	0.56^**^	−	0.81^**^	0.59^**^	0.69^**^	0.26^**^	0.22^**^	−0.14^**^
SS	3.83	0.94	0.10	0.66	0.86	−0.03^**^	−0.02^**^	−0.33^**^	−0.14^**^	−0.01	0.29^**^	0.29^**^	0.18^**^	−0.37^**^	−0.34^**^	0.68^**^	0.67^**^	−	0.69^**^	0.67^**^	0.28^**^	0.23^**^	−0.19^**^
HRP	3.05	0.90	0.23	0.83	0.77	0.02	0.02^**^	−0.35^**^	−0.12^**^	−0.01	0.30^**^	0.32^**^	0.22^**^	−0.30^**^	−0.34^**^	0.55^**^	0.50^**^	0.58^**^	−	0.67^**^	0.39^**^	0.32^**^	−0.03
SC	3.76	0.76	0.16	0.76	0.72	−0.05^**^	−0.03^**^	−0.33^**^	−0.19^**^	−0.06^**^	0.24^**^	0.22^**^	0.17^**^	−0.28^**^	−0.35^**^	0.40^**^	0.49^**^	0.49^**^	0.47^**^	−	0.40^**^	0.27^**^	−0.11^**^
P1M	4.09	0.80	0.15	0.75	0.63	0.11^**^	0.05^**^	−0.15^**^	−0.03^**^	−0.05^**^	0.16^**^	0.20^**^	0.21^**^	−0.25^**^	−0.23^**^	0.20^**^	0.22^**^	0.24^**^	0.23^**^	0.20^**^	−	0.18^**^	−0.03
P2Y	3.14	1.15	0.05	0.48	*r* = 0.82	−0.11^**^	−0.10^**^	−0.06^**^	0.06^**^	0.11^**^	0.21^**^	0.23^**^	0.11^**^	−0.15^**^	−0.06^**^	0.23^**^	0.13^**^	0.18^**^	0.20^**^	0.14^**^	0.13^**^	−	0.19^**^
WLC	2.00	0.77	0.08	0.60	r=0.45	−0.07^**^	−0.12^**^	0.21^**^	0.40^**^	0.22^**^	−0.01	−0.01	−0.07^**^	0.17^**^	0.32^**^	−0.04^**^	−0.21^**^	−0.17^**^	−0.15^**^	−0.24^**^	−0.09^**^	0.00	−

** *p* < 0.01.

ICC, intraclass correlation; TI, turnover intention; QD, quantitative demands; DD, decisional demands; PC, positive challenge; DC, decisional control; CWP, control over work pacing; RA, role ambiguity; RC, role conflict; EL, empowering leadership; FL, fair leadership; SS, support from superior; HRP, human resources primacy; SC, social climate; P1M, predictability next month; P2Y, predictability next 2 years; WLC, work-life conflict.

Response categories were “1 = very seldom or never,” “2 = somewhat seldom,” “3 = sometimes,” “4 = somewhat often,” and “5 = very often or always” for all scales except “predictability during the next 2 years”, “human resource primacy,” and “social climate”: “1 = very little or not at all,” “2 = rather little,” “3 = somewhat,” “4 = rather much,” and “5 = very much.”

### 2.4. Dependent variable—Turnover intention

Turnover intention was measured by two items from the Michigan Organizational Assessment Questionnaire (MOAQ) (Seashore et al., 1982); “I often think about quitting my job” and “It is likely that I will actively look for a new job during the next year,” with response categories on a five point scale ranging from “Completely disagree” to “Completely agree.” The two items were averaged and treated as continuous. The baseline correlation between these items was 0.70 (Table 2).

### 2.5. Control variables

Gender, age, and skill level were included as covariates. Skill levels were determined in accordance with a Norwegian adaptation of the International Standard Classification of Occupations (ISCO-88), by Statistics Norway, expressing educational levels or equivalent levels of work experience typically required for different occupations. T1 turnover intention was included as covariate in prospective analyses. Since intentions to leave may depend on macro-economic fluctuations that affect labor markets, year of measurement was included as a covariate in all analyses.

### 2.6. Statistical analyses

Statistical analyses were run using R version 3.6.1 (R Foundation for Statistical Computing, Vienna, Austria). A strict criterion of statistical significance (*p* < 0.01) was set, due to the high number of tests.

Linear mixed effects regressions were performed, with individual employees nested in work units, using the function “lmer” from the package “lme4” (Bates et al., 2014). These models are equivalent to multilevel models, taking into account possible non-independence of measurements within clusters, correcting for bias due to clustering effects, which may otherwise deflate standard error estimates and increase the risk of Type I error. Also, the possible impact of reporting biases associated with an individual's co-report of work and health are diminished.

Participating organizations differed considerably in size, some being one-unit organizations and others consisting of many work-units distributed over large geographical spaces. Therefore, work-unit membership was considered an appropriate grouping variable, as work-units were assumed to have more in common than the overall organizations.

Prior to the main analyses, attrition analyses were conducted to determine if selective non-response at follow-up could have affected the generalizability of the prospective analyses. This was done by regressing non-response at T2 on the demographic covariates, all predictors and turnover intentions. These analyses were conducted with participants that did respond at T1 and *were also invited at T2*. Additionally, to estimate effects of the study variables on actual turnover, equivalent analyses were conducted to predict *not being invited* at T2. If an employee was invited at T1 but not T2, it would imply that they no longer worked at the same company, i.e., turnover had occurred. However, it should be noted that we do not know in which cases this was voluntary.

**Figure 1:**
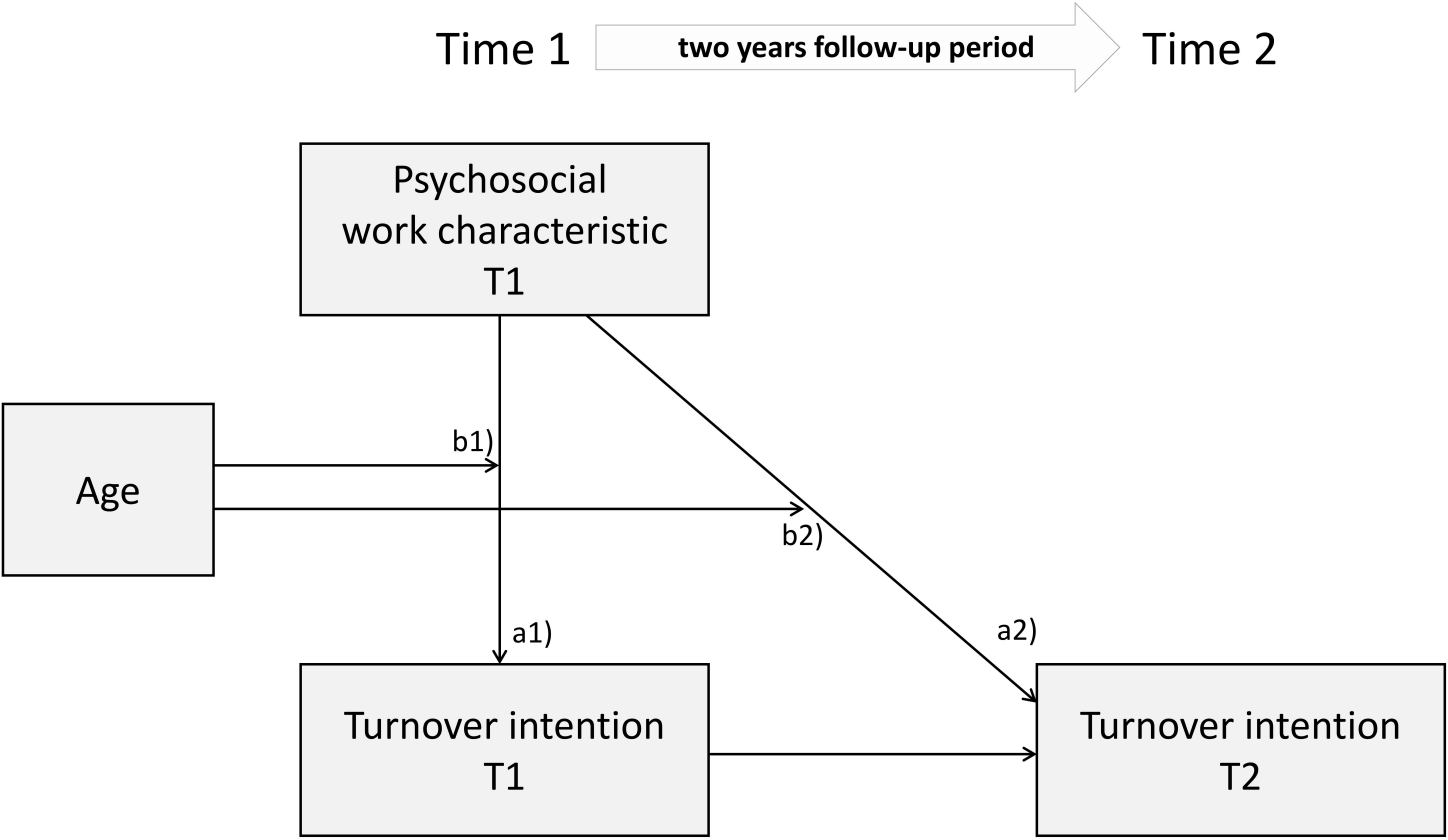
Conceptual overview of the associations tested for the present study. (a1,a2) represent main effects of each studied psychosocial work characteristic, cross-sectionally and prospectively, respectively, and (b1,b2) represent the moderation of the main effects by age.

For the main analyses, regressions were first conducted to determine associations of work characteristics with turnover intention, adjusting for skill level, gender, age, and previous turnover intention. Then, interaction terms were added to investigate potential influences of age on these effects. Figure 1 gives a conceptual overview of the associations tested. Age was treated as continuous, rescaled by dividing by 10, to avoid scaling problems in regression estimations. Age was entered as a predictor along with two product terms modeling the influence of age on the effect of individual level work factors and work-unit level work factors (“cross-level interaction”). For moderated regressions a random slope was added for the level 1 variable (i.e., age), to correctly detect influences of level 2 variables on variation of slopes of level 1 variables (Heisig and Schaeffer, 2019).

To completely remove the influence of correlated reporting biases of exposure and outcome due to individual self-reports (i.e., common method bias), analyses were also conducted with group-level predictors constructed as the mean levels of work factors after exclusion of the individual employee to which they were assigned. Hence, the work factor was in these analyses coworker-reported, operationalized as the mean level of the work factor as reported by employees in the same unit as the individual to which it was assigned.

Work-unit level predictors were constructed by averaging individual responses within work units. Furthermore, to separate effects at different levels as well as to facilitate interpretation of cross-level interaction effects, the individual level predictor was group-mean centered (Enders and Tofighi, 2007). This involves the mean score of each employees' work unit being subtracted from that employee's score. Hence, the shared work-unit variance is removed from individual scores, which become uncorrelated with work-unit means. Hence, effects at individual- and work unit levels are disentangled and can be considered independently of each other (Enders and Tofighi, 2007).

## 3. Results

Table 2 provides cross-sectional means, standard deviations, Cronbach's α, and pairwise correlations for the study variables at the individual and group level.

### 3.1. Attrition analyses

Attrition analyses were conducted for employees that were invited at both time points, i.e., employees that did not leave the company during the follow-up period. Table 3 shows that the probability of attrition was lower with age (OR 0.990, 99% CI 0.983, 0.997) and higher for skill levels “13–15 years” (OR 1.625, 99% CI 1.344, 1.969), “10–12 years” (OR 1.783, 99% CI 1.496, 2.129), and “ < 10 years” (OR 2.429, 99% CI 1.256, 4.572), compared to the skill level “>15 years.” Six of 15 work factors were associated with dropout.

**Table 3:** Actual turnover and attrition.

	**Not responding at T2**			**Not being invited at T2**		
	**(despite being invited)**			**(i.e., no longer employed in the same company)**		
**Predictor**	***N***	**OR**	**99% CI**	***N***	**OR**	**99% CI**
Control variables (mutually adjusted)	7,619			18,632		
Age		0.990^**^	[0.983, 0.997]		0.981^**^	[0.977,0.985]
Female		1.069	[0.934,1.224]		1.267^**^	[1.169,1.373]
Skill level						
>15 years		Ref	−		Ref	−
13–15 years		1.625^**^	[1.344, 1.969]		0.791^**^	[0.710, 0.881]
10–12 years		1.783^**^	[1.496, 2.129]		0.632^**^	[0.572, 0.698]
< 10 years		2.429^**^	[1.256, 4.572]		0.967	[0.663, 1.398]
Managers and unspecified		0.804	[0.592, 1.080]		0.939	[0.801, 1.101]
Turnover intention T1	7,619	1.044	[0.985, 1.105]	12,470	1.297^**^	[1.245, 1.352]
Work factors						
Quantitative demands	7,439	1.105^**^	[1.006, 1.214]	12,176	1.147^**^	[1.070, 1.230]
Decision demands	7,351	1.124^**^	[1.018, 1.242]	12,027	1.010	[0.938, 1.088]
Role ambiguity	7,534	0.911	[0.826, 1.002]	12,326	1.137^**^	[1.062, 1.217]
Role conflict	7,531	0.981	[0.901, 1.068]	12,329	0.955	[0.895, 1.018]
Work-life conflict	7,440	1.081	[0.987, 1.183]	12,194	1.114^**^	[1.042, 1.191]
Decision control	7,228	0.903^**^	[0.819, 0.996]	11,805	0.804^**^	[0.748, 0.865]
Control over work pacing	7,512	0.907^**^	[0.844, 0.975]	12,288	0.903^**^	[0.856, 0.953]
Positive challenge	7,218	0.980	[0.892,1.077]	11,798	0.959	[0.893, 1.030]
Predictability next month	7,538	0.901^**^	[0.827, 0.983]	12,333	0.784^**^	[0.735, 0.836]
Predictability next 2 years	7,162	0.951	[0.895, 1.011]	11,716	1.066^**^	[1.018, 1.117]
Social climate	7,477	0.926	[0.847,1.012]	12,232	0.936	[0.875,1.002]
Human resource primacy	7,274	0.980	[0.907,1.059]	11,913	0.936^**^	[0.883, 0.992]
Empowering leadership	7,532	0.931^**^	[0.871, 0.995]	12,336	0.909^**^	[0.864, 0.955]
Support from superior	7,483	0.956	[0.890, 1.028]	12,267	0.885^**^	[0.839, 0.934]
Fair leadership	7,477	0.938	[0.868, 1.015]	12,201	0.965	[0.910, 1.024]

Separate logistic regressions run for participants that responded to the survey at time 1, predicting (1) not responding at T2 despite being invited (i.e., employed in the company), and (2) not being invited (i.e., not employed in the company) at T2.

** *p* < 0.01.

Regressions with turnover intention or work factors were adjusted for sex, age, and skill level.

### 3.2. Prediction of actual turnover

To strengthen the assumption that the outcome variable of the study reflected an actual intention to change jobs, and to test whether work characteristics were associated with actual turnover, regressions were run with all work factors, covariates, and turnover intention at T1 as predictors of being employed in the same company at T2. This was possible since all employees of each company were invited at each time point. Hence, if a T1 participant was not invited at T2, that employee was no longer employed by that company. This analysis does not distinguish between voluntary turnover and, e.g., layoffs or disability retirement. Nevertheless, the regressions confirmed that employees reporting an intention to leave were more likely to not be employed by the same company 2 years later (OR 1.297, 99% CI 1.245, 1.352, Table 3). Also, 10 out of 15 work factors predicted non-employment at T2, with ORs ranging from 0.784 (99% CI 0.735, 0.836) for predictability next month to 1.147 (99% CI 1.070, 1.230) for quantitative demands (Table 3).

### 3.3. Main analyses

#### 3.3.1. Null model

A “null model” (i.e., intercept only) was run to determine whether adding a random intercept represented a statistically significant improvement over a model with no random effect. A likelihood ratio test supported the tenability of the random intercept model [χ^2^(1) = 220.6, *p* < 0.001, analyses not shown].

Likelihood ratio tests were also run for each separate model for all predictors to compare the random intercept only model with a random slope model. These analyses showed that a random slope improved the model only for decision control [χ^2^(2) = 6.23, *p* = 0.044, analyses not shown]. Hence, random slopes for the main effects were only included for the models with decision control.

#### 3.3.2. Effects of age, sex, and skill level on turnover intention

As shown in Table 4, all the demographic control variables (age, gender, skill level) were associated with turnover cross-sectionally, and age and skill level were prospectively associated with turnover intention. Being female was associated with lower levels of turnover intentions (b = −0.14, *p* < 0.001, cross-sectionally), and higher age was associated with lower turnover intention (b = −0.23, *p* < 0.01, both cross-sectionally and prospectively). Higher skill levels were generally associated with higher turnover intentions.

**Table 4:** Cross-sectional and prospective associations of age, gender, and skill level with turnover intention.

**Predictor**	**b**	**99*%CI***	**b**	**99*%CI***
Gender				
Male	Ref	−	Ref	−
Female	−0.14	[−0.198, −0.078]^**^	−0.04	[−0.112, 0.036]
Skill level				
>15 years	Ref	−	Ref	−
13–15 years	−0.09	[−0.180, −0.002]^*^	−0.03	[−0.145, 0.084]
10–12 years	−0.24	[−0.330, −0.155]^**^	−0.18	[−0.291, −0.076]^**^
< 10 years	−0.19	[−0.496, 0.107]	−0.12	[−0.549, 0.312]
Managers and unspecified	−0.16	[−0.265, −0.048]^**^	−0.09	[−0.220, 0.043]
Age (10 year intervals)	−0.23	[−0.261, −0.207]^**^	−0.23	[−0.266, −0.191]^**^

** *p* < 0.001,

* *p* < 0.01. Regressions were adjusted for year of the survey.

##### 3.3.2.1. Cross-sectional data

All work factors were statistically significantly cross-sectionally associated with turnover intention at baseline, in the hypothesized directions (Table 5). *B*-values ranged from −0.575 (99% CI: −0.613, −0.638) for social climate to 0.439 (99% CI: 0.404, 0.475) for role conflict. The general pattern of associations and statistical significance persisted for work unit level measures of work factors and coworker-reported levels. The exceptions were decision demands and control over work pacing, which were not statistically significant at the group level. The latter was, however, statistically significant when coworker-reported, but the coefficient changed sign, indicating that working in a unit in which coworkers tend to report high control over work pacing is conducive to wanting to leave.

**Table 5:** Cross-sectional data.

		**Individual level**		**Work unit level**		**Coworker-reported**	
**Independent**	***N***	**b**	**99% CI**	**b**	**99% CI**	**b**	**99% CI**
Quantitative demands	12,176	0.239^**^	[0.198, 0.280]	0.256^**^	[0.129, 0.382]	0.249^**^	[0.133, 0.365]
Decision demands	12,027	0.047^*^	[0.005, 0.090]	0.010	[−0.137, 0.157]	0.022	[−0.122, 0.166]
Role ambiguity	12,326	0.411^**^	[0.372, 0.450]	0.602^**^	[0.484, 0.719]	0.593^**^	[0.478,0.708]
Role conflict	12,329	0.439^**^	[0.404, 0.475]	0.578^**^	[0.450, 0.707]	0.536^**^	[0.412,0.660]
Work-life conflict	12,194	0.307^**^	[0.269, 0.344]	0.254^**^	[0.105, 0.403]	0.291^**^	[0.150,0.432]
Decision control	11,805	−0.291^**^	[−0.338, −0.244]	−0.206^**^	[−0.318, −0.094]	−0.196^**^	[−0.306, −0.085]
Control over work pacing	12,288	−0.130^**^	[−0.166, −0.094]	0.060	[−0.003, 0.123]	0.073^*^	[0.012, 0.134]
Positive challenges	11,798	−0.539^**^	[−0.578, −0.500]	−0.615^**^	[−0.740,−0.490]	−0.571^**^	[−0.690, −0.452]
Predictability the next month	12,333	−0.209^**^	[−0.246, −0.171]	−0.324^**^	[−0.446, −0.201]	−0.298^**^	[−0.417, −0.178]
Predictability the next 2 years	11,716	−0.092^**^	[−0.118, −0.066]	−0.139^**^	[−0.246, −0.031]	−0.126^*^	[−0.229,−0.023]
Social climate	12,232	−0.575^**^	[−0.613, −0.538]	−0.611^**^	[−0.720, −0.501]	−0.580^**^	[−0.686, −0.473]
Human resource primacy	11,913	−0.523^**^	[−0.557, −0.490]	−0.457^**^	[−0.539, −0.374]	−0.423^**^	[−0.503, −0.343]
Empowering leadership	12,336	−0.348^**^	[−0.375, −0.320]	−0.369^**^	[−0.463, −0.276]	−0.349^**^	[−0.440, −0.259]
Support from superior	12,267	−0.440^**^	[−0.469, −0.410]	−0.463^**^	[−0.562, −0.363]	−0.445^**^	[−0.542, −0.348]
Fair leadership	12,201	−0.462^**^	[−0.494, −0.429]	−0.531^**^	[−0.638, −0.423]	−0.511^**^	[−0.616, −0.405]

Effect estimates from linear mixed models with psychosocial work factors at the individual- and work unit level as independent variables and turnover intention as dependent variable.

** *p* < 0.001,

* *p* < 0.01.

Regressions were run separately for each independent variable, adjusted for age, gender, skill level, and year of the survey.

Effects of coworker-reported work factors were estimated in separate models including individual level work factors. For brevity, only effects of coworker-reported predictors are shown.

In summary, hypotheses pertaining to associations of work characteristics with turnover intention received support in cross-sectional analyses. Hypotheses regarding job demands (1a) and job control (3a) received only partial support at the group level.

Table 6 shows statistically significant age interactions for 11 of 15 individual level factors, and three factors at the work unit level. Hence, support for individual level interactions was stronger than support for cross-level interactions. However, for quantitative demands (b = −0.047), role conflict (b = −0.064), and predictability for the next month (b = −0.037) interactions were opposite of the hypothesized direction. The same was observed for predictability for the next month (b = −0.115) at the work unit level.

**Table 6:** Cross-sectional data: Interaction effect estimates from linear mixed models with work factors at the individual- and work unit level as independent variables and turnover intention as dependent variable, including age as moderator.

		**Individual level**		**Work unit level**	
**Independent**	***N***	**b**	**99% CI**	**b**	**99% CI**
Quantitative demands	12,176	−0.047^*^	[−0.084, −0.009]	−0.038	[−0.118, 0.041]
Decision demands	12,027	0.013	[−0.026, 0.051]	−0.005	[−0.111, 0.101]
Role ambiguity	12,326	−0.006	[−0.042, 0.031]	−0.007	[−0.091, 0.076]
Role conflict	12,329	−0.064^**^	[−0.097, −0.031]	0.011	[−0.077, 0.099]
Work-life conflict	12,194	−0.012	[−0.047, 0.024]	0.024	[−0.077, 0.124]
Decision control	11,805	0.049^*^	[0.010, 0.089]	0.039	[−0.039, 0.118]
Control over work pacing	12,288	0.006	[−0.028, 0.040]	−0.020	[−0.059, 0.020]
Positive challenges	11,798	0.101^**^	[0.065, 0.137]	0.031	[−0.053, 0.115]
Predictability the next month	12,333	−0.037^*^	[−0.070, −0.003]	−0.115^**^	[−0.199,−0.031]
Predictability the next 2 years	11,716	0.030^*^	[0.007, 0.053]	0.040	[−0.039, 0.118]
Social climate	12,232	0.080^**^	[0.045, 0.116]	0.073	[−0.007,0.154]
Human resource primacy	11913	0.095^**^	[0.063, 0.126]	0.074^*^	[0.015, 0.133]
Empowering leadership	12,336	0.052^**^	[0.026, 0.078]	0.059	[−0.008, 0.127]
Support from superior	12,267	0.073^**^	[0.045, 0.101]	0.063	[−0.013, 0.140]
Fair leadership	12,201	0.072^**^	[0.041, 0.102]	0.055	[−0.028, 0.138]

** *p* < 0.001,

* *p* < 0.01.

Regressions were run separately for each independent variable, adjusted for gender, skill level, and year of the survey.

Overall, the notion that these factors are more influential on older workers received partial support, particularly at the individual level. All hypotheses were partially supported, but different specific factors exhibited interaction effects (e.g., for hypothesis 3b about job control, only decision control was statistically significant). The social and relational factors (social climate, human resource primacy, hypothesis 6b) and leadership styles (support from superior, fair leadership, hypothesis 7b), which can also be considered relational, all exhibited statistically significant interaction effects.

##### 3.3.2.2. Prospective data

Most psychological and social work factors predicted turnover intention 2 years subsequently at the individual level, and seven of 15 predictors were statistically significant also at the work unit level (Table 7). Statistically significant effect estimates at the individual level ranged from −0.126 (99% CI: −0.176, −0.077) for human resource primacy to 0.122 (99% CI: 0.068, 0.176) for role ambiguity. At the work unit level six factors persisted as statistically significant: Role ambiguity, role conflict, empowering leadership, human resource primacy, support from superior, and fair leadership. One factor, social climate, was statistically significant only at the work unit level.

**Table 7:** Prospective data.

		**Individual level**		**Work unit level**		**Coworker-reported**	
**Independent**	***N***	**b**	**99% CI**	**b**	**99% CI**	**b**	**99% CI**
Quantitative demands	5,378	0.062^*^	[0.008, 0.117]	0.093	[−0.070, 0.256]	0.059	[−0.087, 0.204]
Decision demands	5,314	0.019	[−0.037, 0.074]	−0.024	[−0.200, 0.152]	−0.075	[−0.248, 0.098]
Role ambiguity	5,435	0.122^**^	[0.068, 0.176]	0.237^**^	[0.080, 0.394]	0.221^**^	[0.064, 0.377]
Role conflict	5,441	0.106^**^	[0.056, 0.156]	0.197^*^	[0.038, 0.357]	0.164^*^	[0.010, 0.319]
Work-life conflict	5,366	0.041	[−0.009, 0.092]	0.162	[−0.021, 0.345]	0.109	[−0.070, 0.288]
Decision control	5,232	−0.066^*^	[−0.123, −0.008]	0.009	[−0.139, 0.156]	0.041	[−0.106, 0.189]
Control over work pacing	5,431	−0.020	[−0.065, 0.025]	0.070	[−0.012, 0.152]	0.092^*^	[0.015, 0.168]
Positive challenges	5,226	−0.073^**^	[−0.129, −0.017]	−0.141	[−0.300, 0.018]	−0.169^*^	[−0.322, −0.016]
Predictability the next month	5,446	−0.059^*^	[−0.111, −0.007]	−0.057	[−0.205, 0.091]	−0.049	[−0.196,0.099]
Predictability the next 2 years	5,182	−0.015	[−0.048, 0.019]	−0.014	[−0.147, 0.119]	−0.013	[−0.142, 0.116]
Social climate	5,403	−0.052	[−0.108, 0.003]	−0.175^*^	[−0.319, −0.032]	−0.175^*^	[−0.317, −0.034]
Human resource primacy	5,268	−0.126^**^	[−0.176, −0.077]	−0.121^*^	[−0.230, −0.013]	−0.109^*^	[−0.214, −0.005]
Empowering leadership	5,437	−0.074^**^	[−0.112, −0.035]	−0.120^*^	[−0.239, −0.001]	−0.117^*^	[−0.233, −0.001]
Support from superior	5,401	−0.116^**^	[−0.159, −0.073]	−0.145^*^	[−0.275, −0.014]	−0.134^*^	[−0.264, −0.003]
Fair leadership	5,403	−0.094^**^	[−0.141, −0.047]	−0.182^**^	[−0.321, −0.042]	−0.189^**^	[−0.328, −0.050]

Effect estimates from linear mixed models with work factors at the individual- and work unit level at time 1 as independent variables and turnover intention at time 2 as dependent variable.

** *p* < 0.001,

* *p* < 0.01.

Regressions were run separately for each independent variable, adjusted for turnover intention at T1, age, gender, skill level, and year of the survey at T2.

Effects of coworker-reported work factors were estimated in separate models including individual level work factors. For brevity, only effects of coworker-reported predictors are shown.

Results for analyses with work unit mean predictors calculated after “self-exclusion” were similar to those of the conventional multilevel analyses.

Only one interaction effect was statistically significant, for control over work pacing (b = −0.062, 99% CI −0.122, −0.002), (Table 8). Contrary to hypothesis 3b, this suggested the effect to be weaker for older workers.

**Table 8:** Prospective data.

		**Individual level**		**Work unit level**	
**Independent**	**N**	**b**	**99% CI**	**b**	**99% CI**
Quantitative demands	5,378	−0.028	[−0.082, 0.026]	−0.031	[−0.138, 0.075]
Decision demands	5,314	0.027	[−0.027,0.081]	0.023	[−0.133,0.179]
Role ambiguity	5,435	0.006	[−0.046, 0.059]	−0.057	[−0.187, 0.073]
Role conflict	5,441	−0.010	[−0.057, 0.037]	0.068	[−0.060, 0.195]
Work-life conflict	5,366	0.012	[−0.038, 0.062]	−0.004	[−0.160, 0.152]
Decision control	5,232	0.022	[−0.032, 0.075]	−0.077	[−0.189, 0.035]
Control over work pacing	5,431	0.001	[−0.045, 0.046]	−0.062^*^	[−0.122, −0.002]
Positive challenges	5,226	−0.019	[−0.071, 0.034]	−0.018	[−0.143, 0.106]
Predictability the next month	5,446	0.013	[−0.036, 0.061]	−0.031	[−0.150, 0.089]
Predictability the next 2 years	5,182	0.017	[−0.016, 0.049]	0.055	[−0.063, 0.172]
Social climate	5,403	−0.015	[−0.069, 0.039]	−0.007	[−0.129, 0.114]
Human resource primacy	5,268	0.004	[−0.043, 0.051]	−0.036	[−0.116, 0.044]
Empowering leadership	5,437	0.011	[−0.026, 0.048]	0.041	[−0.062, 0.144]
Support from superior	5,401	−0.005	[−0.046, 0.036]	0.019	[−0.099, 0.136]
Fair leadership	5,403	−0.009	[−0.053, 0.036]	0.018	[−0.107, 0.144]

Interaction effect estimates from linear mixed models with work factors at the individual- and work unit level at time 1 as independent variables and turnover intention at time 2 as dependent variable, including age as moderator.

* *p* < 0.01.

Regressions were run separately for each independent variable, adjusted for turnover intention at time 1, gender, skill level, and year of the survey at time 2.

## 4. Discussion

The present results showed that specific psychosocial characteristics of work content and -environment were associated with and predicted turnover and turnover intentions. Most predictors also appeared to be influential at the level of the work unit, strongly suggesting measured work factors that predicted the desire to leave did not merely reflect attributions made by individual employees. Moreover, for employees that were still employed in the same company 2 years after, most of the work characteristics were still associated with a desire to leave.

All hypotheses pertaining to main effects of work factors on turnover received partial support. All factors were associated with turnover intention in some analyses, and in the expected direction. When attempting to design working conditions that motivate employees to remain this is vital information pertaining to specific, modifiable aspects of work that—as opposed to job satisfaction or extraneous circumstances—managers have the power to influence directly.

With regard to the influence of age on the associations between work factors and turnover intention, results were more ambiguous. Interaction at the individual level was observed for most work factors cross-sectionally, but not all in the expected direction. Effects of job demands, role stressors and predictability were *less* rather than more pronounced with age, thereby not supporting hypotheses 1b, 2b, and 5b. Hypotheses 3b, 4b, 6b, and 7b, pertaining to the age moderation of effects of job control, positive challenges, organizational climate, and leadership styles on turnover intentions, were partially supported.

Determining mechanisms which generated the observed associations remains outside the current scope. Several possible pathways are plausible, since each work factor may have specific effects on employee motivation, satisfaction, commitment, and general well-being. The relationship of specific psychological and social work factors with health is well established (see e.g., Christensen and Knardahl, 2010, 2012; Kivimäki et al., 2012; Fishta and Backe, 2015; Kivimäki and Kawachi, 2015; Finne et al., 2016), and it is possible that employees perceive adverse working conditions to affect health, wellbeing, and work ability to the extent that they want to leave their jobs. This could also be a contributing factor to stronger effects in older workers, as they may be generally more prone to develop health problems as a result of poor working conditions. Hence, future studies should examine specific pathways to explain why these work characteristics influence turnover intentions, for instance by modeling health complaints as mediators.

Managers are commonly believed to have moderate direct influence on employee turnover decisions (Allen et al., 2010). The present results suggest empowering-, fair-, and supportive leadership are important to prevent turnover intentions, especially as employees age. Another widely held belief is that a simple one-size-fits-all retention strategy is most effective (Allen et al., 2010). The current results as well as previous studies (Allen et al., 2010) suggest context-specific strategies to be more effective. This highlights the importance of assessing the current state of the organization by surveying employees' appraisal of specific work factors to acquire specific information of working conditions to prevent turnover motivations (Allen et al., 2010).

An important implication of the present findings is that companies should refrain from focusing too narrowly on job satisfaction or limiting their efforts to company perks and monetary rewards that swiftly induce satisfaction. Rather, conscious, systematic efforts should be made to improve work content and -environment in the long term. Firstly, perks may not be as effective as often assumed in inducing job satisfaction (Andersen et al., 2017). Moreover, while low job satisfaction is a salient precursor of turnover intention, and intuitively a useful parameter in work environment surveys, it seems timely to draw more attention to factors that are directly malleable and influence both turnover, motivation, health, and work ability (Emberland and Knardahl, 2015). While surveying job satisfaction may provide snapshots of organizational states that predict turnover, knowledge about specific work factors that—possibly through job satisfaction—influence the desire to leave is more actionable.

Cross-sectional analyses showed that quantitative demands and role conflict exhibited statistically significant *negative* interaction effects, suggesting they become less important for turnover with age. While this does not support our hypothesis that all the studied factors would motivate turnover more strongly with age, it is consistent with previous propositions that older workers are less affected by time pressure demands (unless they are novel demands that need to be accommodated) since they can benefit from crystallized abilities (Abbasi and Bordia, 2019). The same authors also proposed that older workers will be less strongly affected by *choice-based* than *solution-based* role conflicts. That is, role conflicts that require making a choice between conflicting, but distinct options may allow older workers to utilize their crystallized abilities, whereas those that require a more complex solution to be derived will require a higher degree of fluid abilities. The currently employed measurement of role conflict incorporated aspects of both.

The work factors that seemed to be the most consistently associated with turnover intentions, particularly at the work unit level (see Tables 5, 7), were the organizational climate factors, the leadership factors, and role expectations. Interestingly, work unit associations for many of these factors exceeded the individual level associations, suggesting contextual effects that were not as evident at the individual level. For instance, for social climate, the work unit mean was statistically significantly negatively associated with turnover intentions while the individual level association was non-significant. That is, a worker in a unit where coworkers report that the social climate is encouraging, supportive, relaxed and comfortable, and not distrustful and suspicious, is unlikely to intend to leave, although the individual perception of the social climate seems less likely to influence this decision. This pattern was observed for several factors, strengthening the assumption that these factors are indeed important at the level of the work environment, and that they may have effects that are partially independent of the individual perception of them.

Factors of the organizational climate and of leadership were the ones that perhaps most clearly interacted with age, in the hypothesized direction (see Table 6). Leadership styles, particularly fair and supportive leadership, also pertain to relations to employees, hence this lends particular support to the notions derived from SES theory, that socio-emotional goals are more important with age. In other words, a positive, supportive organizational climate may be particularly important in order to retain aging workers. This is consistent with the propositions of Truxillo et al. (2012) regarding social work characteristics and job satisfaction for older workers, as well as the finding of Fazi et al. (2019) that the relationship of interdependence with work engagement was stronger for older workers.

### 4.1. Methodological considerations

Although the sample was large and all employees of the organizations were invited, organizations were not randomly sampled, meaning external validity cannot be defined precisely; we do not know with certainty to whom results apply. Nevertheless, the sample diversity and size suggest results pertain to a fairly general employee population. Also, regardless of sample size selective sampling and response can affect internal validity, i.e., the degree to which observed associations are causal. While higher response rates, as a rule of thumb, are often assumed to improve validity, systematically biased self-selection may hamper internal validity even when response rates are high, while high non-response may not affect internal or external validity if it is random. Therefore, rather than dismissing evidence based on low participation rates, possible reasons for non-response should be considered (Schalm and Kelloway, 2001). If participation is a common consequence of the studied work factors and turnover intention, independently of each other, associations may differ spuriously between respondents and non-respondents (Hernan et al., 2004). In the present data, attrition analyses suggested that while turnover intention predicted non-participation at T2 and thereby not being included in prospective analyses, the choice to participate at T2 among those invited at T2 was not influenced by turnover intention or the studied work factors.

It should also be noted that even if results are valid for the Norwegian world of work, they may not be generalizable to all other countries. The intention to quit, and the actual behavior of quitting, do most likely in most cases rely on a number of precursors, some of which may pertain to the socioeconomic context. For instance, depending on skill levels, the labor market affects the actual intention to quit even when job satisfaction is low. Both socioeconomic and cultural contexts vary, not only between countries, but also over time, it is not possible to specify with certainty for whom and when the present results apply. Nevertheless, it seems reasonable to assume that even when actual intentions to quit do not result, the job characteristics that predict turnover intentions in the current sample should be aversive to most employees under normal conditions.

Prospective analyses are generally recommended to determine the temporal order of independent and dependent variables, but have not been common in turnover research (Rubenstein et al., 2018), and hence represent a strength of the present study. Cross-sectional studies have two important limitations (Spector, 2019): the lack of temporal separation, and—particularly with self-report data—that measurements collected at the same time point from the same source can be tainted by biases that affect independent and dependent measures equally. However, we chose to also include cross-sectional analyses to account for some limitations of *prospective* methods for studying turnover, such as workers with turnover intentions actually leaving the organization during the follow-up period, inducing a selection bias toward the null. Furthermore, the desire to quit may be addressed by job crafting or other resolutions that alleviate turnover intentions.

By utilizing aggregated information at the work unit level, the most important concerns of cross-sectional analyses were also addressed. Additionally, our work-unit level analyses were also conducted with work factor levels reported by *coworkers*. This should eliminate biases caused by independent and dependent variables being reported concurrently by the same individual (i.e., common method bias and reverse causality bias, Podsakoff et al., 2003), as well as ensuring that individuals with high turnover intentions are still retained in the sample.

Some remarks are in order regarding the nature of aggregated measures. They can be viewed as *formative*, meaning that they reflect the average level of a work factor within a unit, without necessarily reflecting the same root phenomenon. In other words, a high average level of a factor (e.g., quantitative demands) does not automatically mean that the source of these high demands is shared between all employees. If one views the aggregated measures in the current study as formative, it implies that work unit effects are not necessarily environmentally shared exposures, but rather the average effect of high levels of a work factor within a unit. If, on the other hand, one views these measures as *reflective* measures, one assumes that they reflect an aspect of the social environment that is not present at the individual level (Lüdtke et al., 2008), but that there is one common, underlying dimension that affects all employees similarly (the “work environment”). For the current study we did not make a distinction between these two, but it should be taken into consideration when interpreting results. Both assumptions imply the diminishing of individual reporting bias, but for certain factors, such as, e.g., social climate, it may seem more reasonable to infer results to the external environment than for others. However, it remains unknown whether the general level of, e.g., role conflict pertained just to a current situation in a work unit or was reflective of a more general aspect of the organization and climate at the respective unit.

As the aim of the current investigation was to assess the effect of each separate work factor on turnover intention, analyses were run separately for each predictor. However, Table 2 demonstrates varying degrees of association between most of the work factors measured. Clearly, there may be complex causal interactions between various factors of the work environment as measured by the QPS_*Nordic*_ (Dallner et al., 2000). The current results do, however, not inform about the precedence of factors with regards to their impact of turnover intention, as they may mediate and moderate the effects of each other. Effect strength or unique contributions, as would be measured by entering all predictors in one regression, may not be appropriate indicators of the real-world impact of a variable when its position on the causal chain from exposure to effect is unknown. Hence, there is a strong need for future studies modeling these causal chains in order to elucidate more specifically the mechanisms at play.

In a large and diverse population there may be a multitude of moderating influences that determine the extent to which various work factors influence turnover intentions. We have investigated the role of age in this respect, but it should be kept in mind that there are many other potential moderators that are poly-relevant, such as gender and occupation. The impact of work characteristics may differ for these and other subgroups, and possibly also the moderating impact of age. Although the large sample of the current study would allow for many such explorations, they remain outside the scope of the study. Future studies should continue to investigate the moderating influences of different factors on the relationship between work characteristics and turnover intentions.

A limitation of the linear regression analyses conducted for the present study is that they assume linear relationships between predictors and turnover intention. For some factors non-linear relationships may be plausible, and recent studies have suggested this to be the case for health effects of factors such as task complexity and time pressure (Sanclemente et al., 2022). In order to detect any obvious non-linearity, we plotted the residuals from the fitted cross-sectional models, and the mean levels of turnover intention, against the levels of the predictors. We did not observe any obvious cases of non-linearity, but it must be noted that thorough explorations of this possibility was beyond the scope of the study and hence we cannot definitively rule out such associations.

Another limitation of the current analyses is that the regressions employed manifest rather than latent variables, meaning they were not corrected statistically for random measurement error. This could imply underestimation of effects. However, the measurement instruments that were used were previously validated and have been demonstrated to have adequate psychometric properties. Nevertheless, the possibility of measurement error should be kept in mind, especially for the factors exhibiting the lowest reliabilities.

Finally, a note on effect magnitudes is in order. Judging whether an effect is “small” or “large” in mixed models is not straightforward, some approximations can be made (Selya et al., 2012). For the present study, the partial *f*^2^ was calculated post hoc for each predictor of the cross-sectional and prospective main effects analyses, using the r package “effectsize” (Ben-Shachar et al., 2020). Based on this, cross-sectionally, only two factors were indicated to have “large” effects, namely individual level decision control and work-unit level organizational commitment. Eight factors exhibited “medium” effects (work-unit levels of human resource primacy, social climate, role ambiguity, fair leadership, positive challenge, support from superior, role conflict, and individual levels of organizational commitment). The remaining effects were either “small” or “very small.” However, such simple classification of magnitude, based on rule of thumb, is problematic since it is unclear what magnitude of effect one should expect when assessing single, specific predictors of complex, multidetermined phenomena. Most likely, the sum and combination of work factors in a work situation has much greater explanatory power in terms of explained variance, but this does not negate the utility of identifying specific, malleable factors that contribute to this variance from different positions of the causal chain from work to turnover intentions.

## 5. Conclusions

The present research should motivate a shift in turnover research from the dominating focus on personal factors and attitudes to environmental characteristics, amenable to practical management in organizations and society. Such knowledge is increasingly crucial to the sustainability of contemporary businesses. The practical implications of the present research are clear, in empirically establishing a broad range of factors that are particularly relevant to human resource practices.

When considering how to retain employees in a workplace it seems to matter whether they experience clarity of and harmony between goals, empowering leadership, and a social climate characterized by high levels of trust, support, and fairness. More generally, being employed in a work unit characterized by high levels of one of those particular factors was associated with not wanting to leave. Finally, with regard to the organizational climate, leadership styles, and positive challenges, this may be particularly true for workers as they get older.

The theoretical implications of the present research are also clear, although no single theory was tested. Research that clarifies the role of characteristics of work and work environment in turnover intentions has been scarce (Kim and Kao, 2014). Comprehensive theories and models of the turnover process should include such factors in the future. Moreover, by bridging turnover research with aging research, the present study highlights the importance of considering boundary conditions such as age when theorizing about turnover as well as work design. Overall, our findings suggest that theories and research of turnover and work design should include greater nuance by investigating multiple factors as well as their differential impact across the life span of workers.

## Data availability statement

The datasets presented in this article are not readily available because the license to collect and store the data from the Data Inspectorate and REK stated specifically that de-identified data will be available only to collaborators with the National Institute of Occupational Health (NIOH) in Norway upon signing a declaration of confidentiality. Thus, data cannot be made publicly available, but may be available upon request. Any requests concerning the availability of the data should be directed to the project leader Jan Emberland, jan.emberland@stami.no or to the general director Margrethe Schøning at margrethe.schøning@stami.no/+47 23 19 51 10 at the National Institute of Occupational Health, Norway. Requests to access the datasets should be directed to Jan Emberland, jan.emberland@stami.no.

## Ethics statement

The studies involving human participants were reviewed and approved by Regional Committees for Medical and Health Research Ethics, Norway (REK). The patients/participants provided their written informed consent to participate in this study.

## Author contributions

JC and SK: conceptualization and writing—review and editing. JC: methodology, software, validation, formal analysis, investigation, data curation, and writing—original draft preparation. SK: resources, supervision, project administration, and funding acquisition. All authors have read and agreed to the published version of the manuscript.

## Funding

This study was funded by the Research Council of Norway (grant number 185209).

## Conflict of interest

The authors declare that the research was conducted in the absence of any commercial or financial relationships that could be construed as a potential conflict of interest.

## Publisher's note

All claims expressed in this article are solely those of the authors and do not necessarily represent those of their affiliated organizations, or those of the publisher, the editors and the reviewers. Any product that may be evaluated in this article, or claim that may be made by its manufacturer, is not guaranteed or endorsed by the publisher.
